# [(3-Methylphenyl)(triphenylphosphonio)methanide-κ*C*]triphenyl­phospho­rane}(penta­fluoro­phenyl-κ*C*)gold(I) diethyl ether solvate

**DOI:** 10.1107/S160053680904152X

**Published:** 2009-10-17

**Authors:** Christoph E. Strasser, Karolien Coetzee, Stephanie Cronje, Helgard G. Raubenheimer

**Affiliations:** a Department of Chemistry and Polymer Science, University of Stellenbosch, Private Bag X1, Matieland 7602, South Africa

## Abstract

The metal atom in the title ylid–gold(I) adduct, [Au(C_6_F_5_)(C_26_H_23_P)]·C_4_H_10_O, exists in a linear coordination environment [C—Au—C = 174.1 (2)°]. The mol­ecule has a short intra­molecular contact involving an aromatic H atom (Au⋯H = 2.64 Å); two adjacent mol­ecules are linked by an Au⋯H_ylid_ inter­action (Au⋯H = 3.14 Å).

## Related literature

For Au⋯H inter­actions, see: Baukova *et al.* (1995 ▶, 1997 ▶), Friedrichs & Jones (2004*a* ▶, 2004*b* ▶, 2004*c* ▶); Räisänen *et al.* (2007 ▶) (Au⋯H inter­actions). For related crystal structures; see: Usón *et al.* (1986 ▶, 1987 ▶, 1990 ▶). For the synthesis of the phospho­rane, see: Friedrich & Henning (1959 ▶); Horner *et al.* (1962 ▶). For the synthesis of the gold reactant and a side-product, see: Usón *et al.* (1989 ▶); Coetzee *et al.* (2007 ▶).
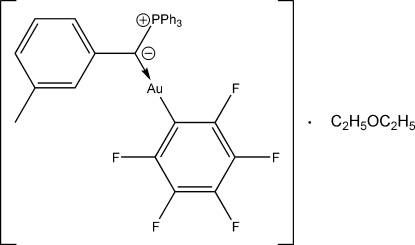


## Experimental

### 

#### Crystal data

[Au(C_6_F_5_)(C_26_H_23_P)]·C_4_H_10_O
*M* *_r_* = 804.56
Orthorhombic, 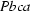

*a* = 21.4958 (10) Å
*b* = 12.4634 (6) Å
*c* = 23.3126 (11) Å
*V* = 6245.7 (5) Å^3^
*Z* = 8
Mo *K*α radiation
μ = 4.82 mm^−1^
*T* = 100 K
0.24 × 0.21 × 0.19 mm

#### Data collection

Bruker APEX CCD area-detector diffractometer
Absorption correction: multi-scan (*SADABS*; Bruker, 2002 ▶) *T*_min_ = 0.328, *T*_max_ = 0.400
37154 measured reflections
7453 independent reflections
5638 reflections with *I* > 2σ(*I*)
*R*_int_ = 0.053

#### Refinement

*R*[*F* ^2^ > 2σ(*F* ^2^)] = 0.037
*wR*(*F* ^2^) = 0.080
*S* = 1.03
7453 reflections
398 parameters
H-atom parameters constrained
Δρ_max_ = 1.85 e Å^−3^
Δρ_min_ = −0.67 e Å^−3^

### 

Data collection: *SMART* (Bruker, 2002 ▶); cell refinement: *SAINT* (Bruker, 2003 ▶); data reduction: *SAINT*; program(s) used to solve structure: *SHELXS97* (Sheldrick, 2008 ▶); program(s) used to refine structure: *SHELXL97* (Sheldrick, 2008 ▶); molecular graphics: *X-SEED* (Atwood & Barbour, 2003 ▶; Barbour, 2001 ▶); software used to prepare material for publication: *X-SEED*.

## Supplementary Material

Crystal structure: contains datablocks I, global. DOI: 10.1107/S160053680904152X/ng2660sup1.cif

Structure factors: contains datablocks I. DOI: 10.1107/S160053680904152X/ng2660Isup2.hkl

Additional supplementary materials:  crystallographic information; 3D view; checkCIF report

## Figures and Tables

**Fig. 1.**
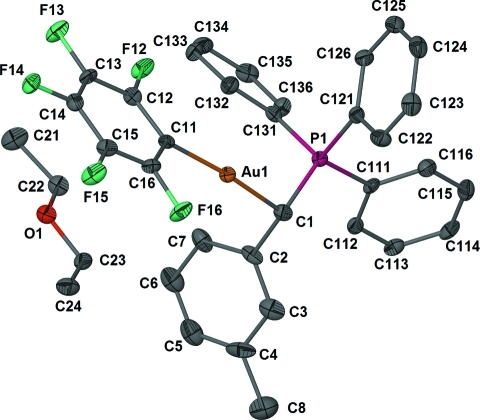
The asymmetric unit of (I), ellipsoids are drawn at the 50% probability level, hydrogen atoms are omitted for clarity.

## supplementary crystallographic information

### Comment

The hydrogen atom H132 of a PPh_3_ phenyl group approaches the gold centre quite closely, the distance of 2.64 Å is at the lower end of the range for Au···H interactions, 2.60 to 3.07 Å, as described by Baukova *et al.* (1995, 1997), Friedrichs & Jones (2004*a*, *b*, *c*) and Räisänen *et al.* (2007). The proton at the ylide carbon atom furthermore links two formula units together that are related by a centre of inversion (symmetry code i = –x, –y+1, –z+1). Another Au···H contact is formed by H23A of the diethyl ether solvent which approaches the gold centre at about 3.04 Å.

Gold(I) ylide complexes thus could represent an interesting field to study Au···H interactions also with other techniques *e.g.* NMR, which has been reported by Baukova *et al.* (1997).

Related compounds to (I) exhibiting similar geometric properties were reported by Usón *et al.* (1986, 1987, 1990). The compound reported in the latest publication also exhibits a Au···H interaction with a separation of 3.08 Å; the molecules are linked to form chains related by 2_1_ screw operations instead of the dimers found in (I).

### Experimental

1,3-Bis[(triphenyl-λ^5^-phosphoranyl)methyl]benzene was prepared according to a modified literature procedure (Friedrich & Henning, 1959; Horner *et al.*, 1962). A suspension of 1,3-bis[(triphenylphosphonio)methyl]benzene(2+) dibromide (0.790 g, 1.00 mmol) and Ag_2_O in a 1:1 ethanol/dichloromethane solvent mixture (60 ml) was stirred for 2 h at room temperature. A suspension of [Au(C_6_F_5_)(tht)] (tht = tetrahydrothiophene; 0.640 g, 1.40 mmol; Usón *et al.*, 1989) in 10 ml of dichloromethane was added to this mixture and stirred for 2 h. The grey suspension was filtered through MgSO_4_ to give a clear, colourless solution. The filtrate was concentrated to dryness yielding a colourless crystalline powder (0.380 g). Crystals of (I) and [Au(C_6_F_5_)(tht)] (Coetzee *et al.*, 2007) suitable for single-crystal X-ray diffraction studies were obtained from a solution of the crude mixture in diethyl ether stored at -10 °C for six days.

### Refinement

All H atoms were positioned geometrically (C—H = 0.95, 1.00 and 0.98 Å for aromatic and aliphatic CH and CH_3_ groups, respectively) and constrained to ride on their parent atoms; *U*_iso_(H) values were set at 1.2 times *U*_eq_(C) for CH groups and 1.5 times *U*_eq_(C) for CH_3_ groups.

The maximum residual electron density of 1.85 e Å^-3^ is located 0.91 Å next to Au1.

### Crystal data

**Table:**

[Au(C_6_F_5_)(C_26_H_23_P)]·C_4_H_10_O	*F*(000) = 3168
*M**_r_* = 804.56	*D*_x_ = 1.711 Mg m^−^^3^
Orthorhombic, *P**b**c**a*	Mo *K*α radiation, λ = 0.71073 Å
Hall symbol: -P 2ac 2ab	Cell parameters from 6992 reflections
*a* = 21.4958 (10) Å	θ = 2.5–27.5°
*b* = 12.4634 (6) Å	µ = 4.82 mm^−^^1^
*c* = 23.3126 (11) Å	*T* = 100 K
*V* = 6245.7 (5) Å^3^	Block, colourless
*Z* = 8	0.24 × 0.21 × 0.19 mm

### Data collection

**Table:**

Bruker APEX CCD area-detector diffractometer	7453 independent reflections
Radiation source: fine-focus sealed tube	5638 reflections with *I* > 2σ(*I*)
graphite	*R*_int_ = 0.053
ω scans	θ_max_ = 28.3°, θ_min_ = 1.8°
Absorption correction: multi-scan (*SADABS*; Bruker, 2002)	*h* = −28→24
*T*_min_ = 0.328, *T*_max_ = 0.400	*k* = −16→11
37154 measured reflections	*l* = −30→30

### Refinement

**Table:**

Refinement on *F*^2^	Primary atom site location: structure-invariant direct methods
Least-squares matrix: full	Secondary atom site location: difference Fourier map
*R*[*F*^2^ > 2σ(*F*^2^)] = 0.037	Hydrogen site location: inferred from neighbouring sites
*wR*(*F*^2^) = 0.080	H-atom parameters constrained
*S* = 1.03	*w* = 1/[σ^2^(*F*_o_^2^) + (0.0346*P*)^2^ + 6.9717*P*] where *P* = (*F*_o_^2^ + 2*F*_c_^2^)/3
7453 reflections	(Δ/σ)_max_ = 0.002
398 parameters	Δρ_max_ = 1.85 e Å^−^^3^
0 restraints	Δρ_min_ = −0.67 e Å^−^^3^

### Special details

**Table:**

Geometry. All e.s.d.'s (except the e.s.d. in the dihedral angle between two l.s. planes) are estimated using the full covariance matrix. The cell e.s.d.'s are taken into account individually in the estimation of e.s.d.'s in distances, angles and torsion angles; correlations between e.s.d.'s in cell parameters are only used when they are defined by crystal symmetry. An approximate (isotropic) treatment of cell e.s.d.'s is used for estimating e.s.d.'s involving l.s. planes.
Refinement. Refinement of *F*^2^ against ALL reflections. The weighted *R*-factor *wR* and goodness of fit *S* are based on *F*^2^, conventional *R*-factors *R* are based on *F*, with *F* set to zero for negative *F*^2^. The threshold expression of *F*^2^ > 2σ(*F*^2^) is used only for calculating *R*-factors(gt) *etc*. and is not relevant to the choice of reflections for refinement. *R*-factors based on *F*^2^ are statistically about twice as large as those based on *F*, and *R*- factors based on ALL data will be even larger.

### Fractional atomic coordinates and isotropic or equivalent isotropic displacement parameters (Å^2^)

**Table:**

	*x*	*y*	*z*	*U*_iso_*/*U*_eq_	
Au1	0.096503 (7)	0.573244 (13)	0.454691 (7)	0.01725 (6)	
P1	0.14663 (5)	0.42332 (9)	0.55425 (4)	0.0171 (2)	
F12	0.21626 (11)	0.7003 (2)	0.40628 (11)	0.0292 (6)	
F13	0.22767 (12)	0.8617 (2)	0.33206 (12)	0.0335 (7)	
F14	0.12514 (12)	0.9566 (2)	0.28610 (11)	0.0249 (6)	
F15	0.01023 (12)	0.8856 (2)	0.31645 (12)	0.0330 (7)	
F16	−0.00304 (11)	0.7257 (2)	0.39183 (11)	0.0310 (6)	
O1	0.11904 (14)	0.5774 (2)	0.26772 (13)	0.0227 (7)	
C1	0.08815 (19)	0.4286 (4)	0.49980 (19)	0.0203 (9)	
H1	0.0469	0.4296	0.5196	0.024*	
C2	0.0881 (2)	0.3335 (4)	0.45888 (19)	0.0249 (10)	
C3	0.0413 (2)	0.2581 (4)	0.4614 (2)	0.0320 (12)	
H3	0.0101	0.2632	0.4902	0.038*	
C4	0.0397 (2)	0.1714 (4)	0.4205 (2)	0.0365 (13)	
C5	0.0861 (2)	0.1670 (5)	0.3799 (2)	0.0372 (13)	
H5	0.0851	0.1106	0.3525	0.045*	
C6	0.1326 (2)	0.2381 (4)	0.3770 (2)	0.0353 (13)	
H6	0.1642	0.2314	0.3487	0.042*	
C7	0.1340 (2)	0.3227 (4)	0.41656 (19)	0.0303 (11)	
H7	0.1667	0.3739	0.4146	0.036*	
C8	−0.0120 (3)	0.0927 (5)	0.4248 (3)	0.0498 (16)	
H8A	−0.0491	0.1219	0.4060	0.075*	
H8B	−0.0211	0.0788	0.4653	0.075*	
H8C	0.0002	0.0256	0.4060	0.075*	
C11	0.10611 (19)	0.7047 (3)	0.40330 (17)	0.0172 (9)	
C12	0.16270 (19)	0.7447 (4)	0.38523 (18)	0.0195 (9)	
C13	0.1703 (2)	0.8267 (4)	0.34713 (18)	0.0205 (9)	
C14	0.1191 (2)	0.8752 (4)	0.32374 (18)	0.0206 (9)	
C15	0.0609 (2)	0.8402 (4)	0.33975 (19)	0.0216 (10)	
C16	0.05562 (19)	0.7576 (4)	0.37831 (18)	0.0207 (9)	
C21	0.2296 (2)	0.5865 (4)	0.2633 (2)	0.0350 (12)	
H21C	0.2683	0.5557	0.2782	0.052*	
H21A	0.2280	0.6632	0.2724	0.052*	
H21B	0.2280	0.5769	0.2216	0.052*	
C22	0.1742 (2)	0.5300 (4)	0.29069 (19)	0.0245 (10)	
H22B	0.1753	0.4523	0.2818	0.029*	
H22A	0.1753	0.5389	0.3329	0.029*	
C23	0.0638 (2)	0.5271 (4)	0.28810 (19)	0.0248 (10)	
H23A	0.0622	0.5301	0.3305	0.030*	
H23B	0.0629	0.4509	0.2761	0.030*	
C24	0.0090 (2)	0.5867 (4)	0.2628 (2)	0.0293 (11)	
H24C	−0.0298	0.5537	0.2761	0.044*	
H24B	0.0110	0.5831	0.2208	0.044*	
H24A	0.0104	0.6619	0.2751	0.044*	
C111	0.14231 (19)	0.3051 (3)	0.59881 (18)	0.0178 (9)	
C112	0.14187 (19)	0.2042 (4)	0.57311 (18)	0.0201 (9)	
H112	0.1441	0.1983	0.5325	0.024*	
C113	0.1381 (2)	0.1128 (4)	0.6065 (2)	0.0230 (10)	
H113	0.1386	0.0442	0.5887	0.028*	
C114	0.1338 (2)	0.1205 (4)	0.6654 (2)	0.0248 (10)	
H114	0.1302	0.0575	0.6881	0.030*	
C115	0.1346 (2)	0.2208 (4)	0.69140 (19)	0.0264 (10)	
H115	0.1322	0.2263	0.7320	0.032*	
C116	0.1388 (2)	0.3131 (4)	0.65806 (19)	0.0237 (10)	
H116	0.1394	0.3816	0.6759	0.028*	
C121	0.1370 (2)	0.5378 (4)	0.60048 (18)	0.0184 (9)	
C122	0.0777 (2)	0.5769 (4)	0.61363 (19)	0.0244 (10)	
H122	0.0421	0.5451	0.5966	0.029*	
C123	0.0709 (2)	0.6616 (4)	0.65137 (19)	0.0276 (11)	
H123	0.0305	0.6876	0.6603	0.033*	
C124	0.1227 (2)	0.7089 (4)	0.67619 (19)	0.0294 (11)	
H124	0.1181	0.7679	0.7016	0.035*	
C125	0.1811 (2)	0.6696 (4)	0.66366 (19)	0.0277 (11)	
H125	0.2167	0.7014	0.6810	0.033*	
C126	0.1887 (2)	0.5845 (4)	0.62621 (18)	0.0226 (10)	
H126	0.2292	0.5579	0.6181	0.027*	
C131	0.22506 (19)	0.4264 (4)	0.52592 (17)	0.0192 (9)	
C132	0.2439 (2)	0.5107 (4)	0.49073 (18)	0.0231 (10)	
H132	0.2156	0.5670	0.4819	0.028*	
C133	0.3034 (2)	0.5126 (4)	0.46867 (19)	0.0258 (11)	
H133	0.3156	0.5695	0.4440	0.031*	
C134	0.3454 (2)	0.4325 (4)	0.4821 (2)	0.0259 (10)	
H134	0.3863	0.4345	0.4667	0.031*	
C135	0.3276 (2)	0.3490 (4)	0.5181 (2)	0.0268 (11)	
H135	0.3564	0.2942	0.5279	0.032*	
C136	0.2679 (2)	0.3461 (4)	0.53952 (18)	0.0228 (10)	
H136	0.2557	0.2888	0.5639	0.027*	

### Atomic displacement parameters (Å^2^)

**Table:**

	*U*^11^	*U*^22^	*U*^33^	*U*^12^	*U*^13^	*U*^23^
Au1	0.01701 (9)	0.01638 (9)	0.01837 (9)	0.00076 (7)	−0.00176 (6)	0.00163 (7)
P1	0.0167 (5)	0.0165 (5)	0.0182 (5)	0.0004 (4)	−0.0008 (4)	0.0008 (5)
F12	0.0152 (13)	0.0308 (16)	0.0415 (16)	0.0013 (11)	−0.0021 (11)	0.0149 (13)
F13	0.0204 (14)	0.0361 (17)	0.0440 (17)	−0.0037 (12)	0.0033 (12)	0.0152 (14)
F14	0.0281 (14)	0.0190 (14)	0.0276 (14)	−0.0012 (11)	0.0025 (11)	0.0099 (11)
F15	0.0203 (14)	0.0330 (16)	0.0457 (17)	0.0007 (12)	−0.0079 (12)	0.0189 (14)
F16	0.0161 (13)	0.0355 (16)	0.0415 (16)	−0.0047 (12)	−0.0013 (11)	0.0166 (14)
O1	0.0248 (16)	0.0184 (16)	0.0249 (16)	0.0031 (13)	−0.0042 (13)	0.0023 (14)
C1	0.018 (2)	0.021 (2)	0.023 (2)	0.0021 (19)	−0.0045 (16)	0.0004 (19)
C2	0.038 (3)	0.013 (2)	0.023 (2)	0.0031 (19)	−0.013 (2)	−0.0001 (19)
C3	0.035 (3)	0.023 (3)	0.039 (3)	0.008 (2)	−0.014 (2)	0.001 (2)
C4	0.033 (3)	0.023 (3)	0.053 (3)	−0.005 (2)	−0.026 (3)	0.005 (3)
C5	0.038 (3)	0.044 (3)	0.030 (3)	0.016 (3)	−0.008 (2)	−0.003 (2)
C6	0.034 (3)	0.040 (3)	0.032 (3)	0.014 (3)	−0.008 (2)	−0.003 (2)
C7	0.038 (3)	0.029 (3)	0.023 (2)	0.011 (2)	−0.008 (2)	−0.002 (2)
C8	0.045 (4)	0.045 (4)	0.060 (4)	−0.010 (3)	0.000 (3)	0.000 (3)
C11	0.019 (2)	0.015 (2)	0.018 (2)	−0.0028 (17)	−0.0018 (16)	−0.0026 (17)
C12	0.017 (2)	0.019 (2)	0.022 (2)	0.0065 (18)	−0.0032 (17)	0.0010 (18)
C13	0.015 (2)	0.021 (2)	0.025 (2)	−0.0046 (18)	0.0034 (17)	0.0007 (19)
C14	0.028 (2)	0.013 (2)	0.020 (2)	−0.0008 (19)	−0.0007 (18)	0.0010 (18)
C15	0.018 (2)	0.019 (2)	0.028 (2)	0.0034 (18)	−0.0064 (18)	0.0010 (19)
C16	0.016 (2)	0.020 (2)	0.025 (2)	−0.0031 (18)	−0.0018 (17)	−0.0019 (19)
C21	0.024 (3)	0.028 (3)	0.053 (3)	0.005 (2)	−0.002 (2)	0.009 (3)
C22	0.026 (2)	0.019 (2)	0.028 (2)	0.006 (2)	−0.004 (2)	0.002 (2)
C23	0.027 (3)	0.022 (2)	0.024 (2)	−0.007 (2)	0.0041 (19)	−0.003 (2)
C24	0.022 (2)	0.031 (3)	0.035 (3)	−0.001 (2)	−0.004 (2)	−0.005 (2)
C111	0.016 (2)	0.015 (2)	0.022 (2)	−0.0023 (17)	0.0001 (17)	0.0072 (18)
C112	0.018 (2)	0.023 (2)	0.020 (2)	−0.0008 (19)	0.0009 (17)	0.0016 (19)
C113	0.017 (2)	0.020 (2)	0.032 (3)	−0.0004 (18)	0.0015 (18)	0.002 (2)
C114	0.019 (2)	0.020 (2)	0.035 (3)	−0.0006 (19)	0.0027 (19)	0.012 (2)
C115	0.028 (3)	0.029 (3)	0.022 (2)	0.001 (2)	0.0016 (19)	0.006 (2)
C116	0.023 (2)	0.022 (2)	0.026 (2)	−0.0012 (19)	0.0022 (18)	−0.0012 (19)
C121	0.020 (2)	0.018 (2)	0.017 (2)	0.0005 (18)	−0.0006 (17)	0.0026 (17)
C122	0.019 (2)	0.027 (3)	0.027 (2)	0.002 (2)	−0.0063 (18)	−0.003 (2)
C123	0.032 (3)	0.023 (3)	0.028 (3)	0.009 (2)	0.004 (2)	−0.001 (2)
C124	0.046 (3)	0.019 (3)	0.023 (2)	−0.001 (2)	0.004 (2)	−0.003 (2)
C125	0.033 (3)	0.025 (3)	0.025 (2)	−0.008 (2)	0.001 (2)	−0.004 (2)
C126	0.019 (2)	0.022 (2)	0.026 (2)	−0.0018 (19)	0.0013 (17)	−0.003 (2)
C131	0.021 (2)	0.020 (2)	0.0165 (19)	0.0008 (19)	−0.0022 (16)	−0.0042 (19)
C132	0.018 (2)	0.027 (3)	0.024 (2)	0.0000 (19)	−0.0022 (18)	0.005 (2)
C133	0.022 (2)	0.032 (3)	0.024 (2)	−0.005 (2)	0.0027 (18)	0.003 (2)
C134	0.019 (2)	0.029 (3)	0.030 (2)	−0.003 (2)	0.0031 (18)	−0.008 (2)
C135	0.020 (2)	0.025 (3)	0.035 (3)	0.007 (2)	−0.002 (2)	−0.007 (2)
C136	0.021 (2)	0.023 (2)	0.024 (2)	0.0021 (18)	0.0013 (18)	0.003 (2)

### Geometric parameters (Å, °)

**Table:**

Au1—C11	2.040 (4)	C22—H22B	0.9900
Au1—C1	2.094 (4)	C22—H22A	0.9900
Au1—H132	2.6396	C23—C24	1.511 (6)
Au1—H1^i^	3.1400	C23—H23A	0.9900
P1—C1	1.788 (4)	C23—H23B	0.9900
P1—C121	1.800 (5)	C24—H24C	0.9800
P1—C111	1.805 (4)	C24—H24B	0.9800
P1—C131	1.811 (4)	C24—H24A	0.9800
F12—C12	1.368 (5)	C111—C116	1.387 (6)
F13—C13	1.355 (5)	C111—C112	1.392 (6)
F14—C14	1.348 (5)	C112—C113	1.382 (6)
F15—C15	1.342 (5)	C112—H112	0.9500
F16—C16	1.359 (5)	C113—C114	1.381 (6)
O1—C23	1.425 (5)	C113—H113	0.9500
O1—C22	1.430 (5)	C114—C115	1.388 (6)
C1—C2	1.522 (6)	C114—H114	0.9500
C1—H1	1.0000	C115—C116	1.392 (6)
C2—C3	1.378 (7)	C115—H115	0.9500
C2—C7	1.401 (7)	C116—H116	0.9500
C3—C4	1.442 (7)	C121—C126	1.390 (6)
C3—H3	0.9500	C121—C122	1.399 (6)
C4—C5	1.375 (7)	C122—C123	1.382 (6)
C4—C8	1.486 (7)	C122—H122	0.9500
C5—C6	1.337 (8)	C123—C124	1.387 (7)
C5—H5	0.9500	C123—H123	0.9500
C6—C7	1.401 (7)	C124—C125	1.379 (7)
C6—H6	0.9500	C124—H124	0.9500
C7—H7	0.9500	C125—C126	1.383 (6)
C8—H8A	0.9800	C125—H125	0.9500
C8—H8B	0.9800	C126—H126	0.9500
C8—H8C	0.9800	C131—C132	1.393 (6)
C11—C12	1.380 (6)	C131—C136	1.396 (6)
C11—C16	1.397 (6)	C132—C133	1.379 (6)
C12—C13	1.364 (6)	C132—H132	0.9500
C13—C14	1.368 (6)	C133—C134	1.382 (7)
C14—C15	1.377 (6)	C133—H133	0.9500
C15—C16	1.371 (6)	C134—C135	1.391 (7)
C21—C22	1.522 (6)	C134—H134	0.9500
C21—H21C	0.9800	C135—C136	1.376 (6)
C21—H21A	0.9800	C135—H135	0.9500
C21—H21B	0.9800	C136—H136	0.9500
			
C11—Au1—C1	174.05 (16)	O1—C22—H22A	110.2
C11—Au1—H132	93.8	C21—C22—H22A	110.2
C1—Au1—H132	86.4	H22B—C22—H22A	108.5
C11—Au1—H1^i^	102.7	O1—C23—C24	107.6 (4)
C1—Au1—H1^i^	79.1	O1—C23—H23A	110.2
H132—Au1—H1^i^	155.0	C24—C23—H23A	110.2
C1—P1—C121	108.4 (2)	O1—C23—H23B	110.2
C1—P1—C111	113.7 (2)	C24—C23—H23B	110.2
C121—P1—C111	107.3 (2)	H23A—C23—H23B	108.5
C1—P1—C131	113.3 (2)	C23—C24—H24C	109.5
C121—P1—C131	108.0 (2)	C23—C24—H24B	109.5
C111—P1—C131	106.0 (2)	H24C—C24—H24B	109.5
C23—O1—C22	112.6 (3)	C23—C24—H24A	109.5
C2—C1—P1	114.6 (3)	H24C—C24—H24A	109.5
C2—C1—Au1	110.8 (3)	H24B—C24—H24A	109.5
P1—C1—Au1	109.2 (2)	C116—C111—C112	119.6 (4)
C2—C1—H1	107.3	C116—C111—P1	121.1 (3)
P1—C1—H1	107.3	C112—C111—P1	119.3 (3)
Au1—C1—H1	107.3	C113—C112—C111	120.2 (4)
C3—C2—C7	118.6 (4)	C113—C112—H112	119.9
C3—C2—C1	120.3 (4)	C111—C112—H112	119.9
C7—C2—C1	121.0 (4)	C114—C113—C112	120.4 (4)
C2—C3—C4	120.0 (5)	C114—C113—H113	119.8
C2—C3—H3	120.0	C112—C113—H113	119.8
C4—C3—H3	120.0	C113—C114—C115	119.7 (4)
C5—C4—C3	117.9 (5)	C113—C114—H114	120.1
C5—C4—C8	124.3 (5)	C115—C114—H114	120.1
C3—C4—C8	117.8 (5)	C114—C115—C116	120.1 (4)
C6—C5—C4	123.4 (5)	C114—C115—H115	120.0
C6—C5—H5	118.3	C116—C115—H115	120.0
C4—C5—H5	118.3	C111—C116—C115	120.0 (4)
C5—C6—C7	118.8 (5)	C111—C116—H116	120.0
C5—C6—H6	120.6	C115—C116—H116	120.0
C7—C6—H6	120.6	C126—C121—C122	119.2 (4)
C6—C7—C2	121.4 (5)	C126—C121—P1	119.9 (3)
C6—C7—H7	119.3	C122—C121—P1	120.8 (3)
C2—C7—H7	119.3	C123—C122—C121	120.2 (4)
C4—C8—H8A	109.5	C123—C122—H122	119.9
C4—C8—H8B	109.5	C121—C122—H122	119.9
H8A—C8—H8B	109.5	C122—C123—C124	120.3 (5)
C4—C8—H8C	109.5	C122—C123—H123	119.9
H8A—C8—H8C	109.5	C124—C123—H123	119.9
H8B—C8—H8C	109.5	C125—C124—C123	119.5 (4)
C12—C11—C16	112.8 (4)	C125—C124—H124	120.3
C12—C11—Au1	123.9 (3)	C123—C124—H124	120.3
C16—C11—Au1	123.0 (3)	C124—C125—C126	120.9 (4)
C13—C12—F12	115.9 (4)	C124—C125—H125	119.6
C13—C12—C11	125.0 (4)	C126—C125—H125	119.6
F12—C12—C11	119.1 (4)	C125—C126—C121	120.0 (4)
F13—C13—C12	121.2 (4)	C125—C126—H126	120.0
F13—C13—C14	119.1 (4)	C121—C126—H126	120.0
C12—C13—C14	119.7 (4)	C132—C131—C136	118.9 (4)
F14—C14—C13	121.0 (4)	C132—C131—P1	120.1 (3)
F14—C14—C15	120.2 (4)	C136—C131—P1	121.1 (3)
C13—C14—C15	118.9 (4)	C133—C132—C131	120.1 (4)
F15—C15—C16	121.0 (4)	C133—C132—H132	120.0
F15—C15—C14	119.6 (4)	C131—C132—H132	120.0
C16—C15—C14	119.4 (4)	C132—C133—C134	120.7 (4)
F16—C16—C15	116.6 (4)	C132—C133—H133	119.7
F16—C16—C11	119.1 (4)	C134—C133—H133	119.7
C15—C16—C11	124.3 (4)	C133—C134—C135	119.8 (4)
C22—C21—H21C	109.5	C133—C134—H134	120.1
C22—C21—H21A	109.5	C135—C134—H134	120.1
H21C—C21—H21A	109.5	C136—C135—C134	119.7 (4)
C22—C21—H21B	109.5	C136—C135—H135	120.2
H21C—C21—H21B	109.5	C134—C135—H135	120.2
H21A—C21—H21B	109.5	C135—C136—C131	120.9 (4)
O1—C22—C21	107.4 (4)	C135—C136—H136	119.5
O1—C22—H22B	110.2	C131—C136—H136	119.5
C21—C22—H22B	110.2		
			
C121—P1—C1—C2	−177.0 (3)	C12—C11—C16—F16	179.3 (4)
C111—P1—C1—C2	−57.8 (4)	Au1—C11—C16—F16	5.0 (6)
C131—P1—C1—C2	63.2 (4)	C12—C11—C16—C15	0.3 (6)
C121—P1—C1—Au1	58.0 (3)	Au1—C11—C16—C15	−174.1 (3)
C111—P1—C1—Au1	177.18 (19)	C23—O1—C22—C21	177.0 (4)
C131—P1—C1—Au1	−61.8 (3)	C22—O1—C23—C24	177.8 (4)
H132—Au1—C1—C2	−99.3	C1—P1—C111—C116	−125.8 (4)
H1^i^—Au1—C1—C2	101.2	C121—P1—C111—C116	−6.0 (4)
H132—Au1—C1—P1	27.9	C131—P1—C111—C116	109.1 (4)
H1^i^—Au1—C1—P1	−131.6	C1—P1—C111—C112	53.6 (4)
P1—C1—C2—C3	108.3 (4)	C121—P1—C111—C112	173.4 (3)
Au1—C1—C2—C3	−127.6 (4)	C131—P1—C111—C112	−71.5 (4)
P1—C1—C2—C7	−74.1 (5)	C116—C111—C112—C113	−0.1 (6)
Au1—C1—C2—C7	50.0 (5)	P1—C111—C112—C113	−179.5 (3)
C7—C2—C3—C4	−1.0 (7)	C111—C112—C113—C114	1.1 (6)
C1—C2—C3—C4	176.6 (4)	C112—C113—C114—C115	−1.5 (7)
C2—C3—C4—C5	0.4 (7)	C113—C114—C115—C116	1.0 (7)
C2—C3—C4—C8	−179.5 (5)	C112—C111—C116—C115	−0.4 (6)
C3—C4—C5—C6	0.7 (8)	P1—C111—C116—C115	179.0 (3)
C8—C4—C5—C6	−179.4 (5)	C114—C115—C116—C111	0.0 (7)
C4—C5—C6—C7	−1.1 (8)	C1—P1—C121—C126	−147.8 (4)
C5—C6—C7—C2	0.5 (7)	C111—P1—C121—C126	89.0 (4)
C3—C2—C7—C6	0.6 (7)	C131—P1—C121—C126	−24.7 (4)
C1—C2—C7—C6	−177.0 (4)	C1—P1—C121—C122	36.0 (4)
H132—Au1—C11—C12	12.5	C111—P1—C121—C122	−87.2 (4)
H1^i^—Au1—C11—C12	173.6	C131—P1—C121—C122	159.1 (4)
H132—Au1—C11—C16	−173.8	C126—C121—C122—C123	0.7 (7)
H1^i^—Au1—C11—C16	−12.7	P1—C121—C122—C123	176.9 (4)
C16—C11—C12—C13	−0.1 (6)	C121—C122—C123—C124	0.4 (7)
Au1—C11—C12—C13	174.2 (3)	C122—C123—C124—C125	−1.0 (7)
C16—C11—C12—F12	180.0 (4)	C123—C124—C125—C126	0.7 (7)
Au1—C11—C12—F12	−5.7 (6)	C124—C125—C126—C121	0.3 (7)
F12—C12—C13—F13	−0.7 (6)	C122—C121—C126—C125	−1.0 (6)
C11—C12—C13—F13	179.4 (4)	P1—C121—C126—C125	−177.3 (3)
F12—C12—C13—C14	179.6 (4)	C1—P1—C131—C132	56.4 (4)
C11—C12—C13—C14	−0.3 (7)	C121—P1—C131—C132	−63.6 (4)
F13—C13—C14—F14	0.1 (6)	C111—P1—C131—C132	−178.2 (3)
C12—C13—C14—F14	179.8 (4)	C1—P1—C131—C136	−124.7 (4)
F13—C13—C14—C15	−179.2 (4)	C121—P1—C131—C136	115.3 (4)
C12—C13—C14—C15	0.5 (7)	C111—P1—C131—C136	0.7 (4)
F14—C14—C15—F15	1.9 (6)	C136—C131—C132—C133	1.7 (6)
C13—C14—C15—F15	−178.8 (4)	P1—C131—C132—C133	−179.4 (3)
F14—C14—C15—C16	−179.7 (4)	C131—C132—C133—C134	−1.4 (7)
C13—C14—C15—C16	−0.4 (7)	C132—C133—C134—C135	0.1 (7)
F15—C15—C16—F16	−0.7 (6)	C133—C134—C135—C136	0.8 (7)
C14—C15—C16—F16	−179.1 (4)	C134—C135—C136—C131	−0.5 (7)
F15—C15—C16—C11	178.4 (4)	C132—C131—C136—C135	−0.8 (6)
C14—C15—C16—C11	0.0 (7)	P1—C131—C136—C135	−179.7 (3)

Symmetry codes: (i) −*x*, −*y*+1, −*z*+1.
